# First Reported Case of *Cryptococcus gattii* in the Southeastern USA: Implications for Travel-Associated Acquisition of an Emerging Pathogen

**DOI:** 10.1371/journal.pone.0005851

**Published:** 2009-06-10

**Authors:** Edmond J. Byrnes, Wenjun Li, Yonathan Lewit, John R. Perfect, Dee A. Carter, Gary M. Cox, Joseph Heitman

**Affiliations:** 1 Department of Molecular Genetics and Microbiology, Duke University Medical Center, Durham, North Carolina, United States of America; 2 Department of Medicine, Duke University Medical Center, Durham, North Carolina, United States of America; 3 Department of Molecular and Microbial Biosciences, The University of Sydney, Sydney, New South Wales, Australia; Harvard University, United States of America

## Abstract

In 2007, the first confirmed case of *Cryptococcus gattii* was reported in the state of North Carolina, USA. An otherwise healthy HIV negative male patient presented with a large upper thigh cryptococcoma in February, which was surgically removed and the patient was started on long-term high-dose fluconazole treatment. In May of 2007, the patient presented to the Duke University hospital emergency room with seizures. Magnetic resonance imaging revealed two large CNS lesions found to be cryptococcomas based on brain biopsy. Prior chest CT imaging had revealed small lung nodules indicating that *C. gattii* spores or desiccated yeast were likely inhaled into the lungs and dissemination occurred to both the leg and CNS. The patient's travel history included a visit throughout the San Francisco, CA region in September through October of 2006, consistent with acquisition during this time period. Cultures from both the leg and brain biopsies were subjected to analysis. Based on phenotypic and molecular methods, both isolates were *C. gattii,* VGI molecular type, and distinct from the Vancouver Island outbreak isolates. Based on multilocus sequence typing of coding and noncoding regions and virulence in a heterologous host model, the leg and brain isolates are identical, but the two differed in mating fertility. Two clinical isolates, one from a transplant recipient in San Francisco and the other from Australia, were identical to the North Carolina clinical isolate at all markers tested. Closely related isolates that differ at only one or a few noncoding markers are present in the Australian environment. Taken together, these findings support a model in which *C. gattii* VGI was transferred from Australia to California, possibly though an association with its common host plant *E. camaldulensis,* and the patient was exposed in San Francisco and returned to present with disease in North Carolina.

## Introduction

Significant medical advances have been achieved in the fields of antimicrobial agents and vaccine development, yet both newly emerging and re-emerging infectious diseases in humans, livestock, and plants remain serious global health and economic burdens [1], [2]. Several factors influence the emergence and re-emergence of infectious diseases, and two are globalization and an increasing population of immunocompromised hosts [3], [4]. These have significantly impacted the emergence of systemic fungal infections over the past two decades, largely due to widespread use of broad-spectrum antibiotics, advances in healthcare, and the HIV pandemic [5].

An essential component for tracking emergence and epidemiology of bacterial, parasitic, and fungal infections is molecular strain typing. In the genomic era, whole genome sequences have allowed comprehensive typing though sequence-based methods, including multilocus sequence and variable number of tandem repeat (VNTR) typing approaches [6], [7]. Each method has distinct benefits for increasing typing resolution, and can be used concomitantly to increase the overall power of molecular typing. Multilocus sequence typing has been widely applied to the fungal kingdom, and in particular for the *Cryptococcus* species complex [8], [9], [10], [11]. Fewer studies have applied VNTR analysis to fungal pathogens, which was developed and is widely used in studies of bacterial pathogens [7], [12], [13], [14]. We present here the first application of a combined MLST/VNTR approach to establish relationships among a group of emerging *Cryptococcus gattii* clinical and environmental isolates.

*C. gattii* is a basidiomycetous yeast closely related to other members of the *Cryptococcus* pathogenic species complex, including *C. neoformans* var. *grubii* and var. *neoformans* [15], [16], [17]. *C. gattii* has often been associated with tropical and subtropical climates including Australia and South America [15], and has emerged as a fungal pathogen of humans and animals in temperate climates including Vancouver Island, mainland British Columbia, Canada, and the United States Pacific Northwest, including Washington and Oregon [10], [18], [19], [20], [21], [22], [23]. The species *C. gattii* can be subdivided into serotypes B and C based on unique capsular antigenic determinants [24], [25]. In addition, the species can be divided into four molecular types (VGI, VGII, VGIII, VGIV) based on evidence from Amplified Fragment Length Polymorphisms (AFLP), Random Amplification of Polymorphic DNA (RAPD), and Multilocus Sequence Typing (MLST) [10], [11], [26]. Genetic exchange between the four VG molecular types is rare, indicating that these likely represent cryptic species [10], [11].

Cryptococcosis is the disease caused by the pathogenic *Cryptococcus* species complex and usually results in pulmonary infection/pneumonia, central nervous system (CNS) dissemination, and in some cases cryptococcoma formation [17], [27]. *C. neoformans* and *C. gattii* share some common virulence attributes but are also distinct. *C. neoformans* has a global distribution and predominantly infects immunosuppressed hosts, while *C. gattii* is more geographically restricted and commonly infects immunocompetent individuals [28]. Incidence in immunocompetent individuals is particularly high within the VGI and VGII molecular types. Molecular type VGI commonly causes infections in Australia, whereas the VGII molecular type is responsible for the vast majority of cases related to the Vancouver Island outbreak, and its expansion into the North American Pacific NW [17], [19], [29]. The VGIII and VGIV molecular types have been reported to infect immunocompromised patients, including HIV/AIDS patients and organ transplant recipients [30], [31], [32].

In the United States and Canada, a major focus of *C. gattii* research has been on molecular type VGII due to the high percentage (∼95%) of clinical cases caused by this molecular type in otherwise healthy individuals, while only ∼5% of cases result from VGI infection [8], [19], [20]. While VGI is the most common infectious molecular type globally and has been reported in cryptococcosis cases from the Americas including Canada, Mexico, and South America, the overall number of isolates from the United States has been low [10], [17]. In the United States there have been two confirmed cases of *C. gattii* molecular type VGI, both in California. One case occurred in a male Atlantic bottlenose dolphin (*Tursiops truncatus*) in San Diego; the other was in a liver transplant recipient in San Francisco [32], [33]. The isolates from these cases differed based on multilocus sequence type (MLST) analysis, but both are closely related or identical to isolates from Australia, indicating that an environmental source in California may have originated from the large number of *Eucalyptus* trees imported from Australia to California, particularly due to the strong environmental association between the VGI molecular type and *Eucalyptus* trees [10], [34].

*C. gattii* has never been isolated from environmental sources in the Eastern United States [35], and until the present study no cases in the region have been reported in humans or animals. In this study we present the first reported clinical case of *C. gattii* in the Southeastern United States, which occurred in an immunocompetent individual residing in central North Carolina. We show that this case, which resulted in large cryptococcomal granulomas in the leg and brain, resulted from infection with a *C. gattii* VGI type isolate that is distinct from both the common VGII and rarer VGI isolates associated with the Vancouver Island/Pacific NW *C. gattii* outbreak. The molecular genotype of the isolates from both leg and brain biopsies show that the molecular profile based on multilocus sequence (MLS) analysis of both coding (MLST) and more variable noncoding (VNTR) markers is shared with only two clinical isolates (one from Australia, and the other from an organ transplant recipient in San Francisco, California) out of 85 VGI strains typed [10], [32]. While this is the first reported case in the Eastern United States, the suspected ecological niche is in the Western United States, and the patient's travel history is consistent with acquisition in the San Francisco metropolitan area. Travel to endemic areas is known to increase risk for *C. gattii* exposure and disease, and leads to acquisition in one region with presentation in a distant location [36], [37]. The Australian case, California transplant patient, and North Carolina patient isolates are closely related to, but not identical at all noncoding genomic markers (VNTR) with Australian environmental isolates. Therefore, the Australian environment is the most likely source of the original isolates, and additional sampling may be necessary, as subtle genetic changes may have occurred during expansion or infection. Our results further support the well documented emergence of *C. gattii* in temperate climates in the Western United States, and put forth a model in which an emergence from Australia to California has resulted in travel-associated disease presentation in the Southeastern United States.

## Materials and Methods

### Isolate Identification

Melanin production was assayed by growth and the production of dark pigmentation on Staib niger seed agar medium, and urease activity was detected by growth and alkaline pH change on Christensen's Agar. These tests established that isolates were either *C. neoformans* or *C. gattii*. Isolates were then examined for resistance to canavanine and utilization of glycine on L-canavanine, glycine, 2-bromothymol blue, (CGB) agar. Growth on CGB agar indicates that isolates are canavanine resistant, and able to use glycine as a sole carbon source, triggering a bromothymol blue color reaction indicative of *C. gattii*, whereas *C. neoformans* is sensitive to canavanine, and cannot use glycine as a sole carbon source, resulting in no growth or coloration on this selective indicator medium. Capsule identification was conducted though India ink analysis and microscopy after growth on Dulbecco's Modified Eagle's Medium (DMEM) for 72 hours at 37°C.

### Molecular Typing

For multilocus sequence typing analysis (MLST), each isolate was analyzed with a minimum of eight and in some cases eleven unlinked loci [6], [9], [10]. For each isolate, genomic DNA was extracted using the MasterPure™ yeast DNA purification kit (Epicentre Biotechnologies), PCR amplified, purified and sequenced. All primers used for the analysis were designed specifically to amplify open reading frame (ORF) gene sequence regions including those with non-coding DNA regions to maximize discriminatory power (Table S1). All PCR products were sequenced, and novel sequences were re-amplified and sequenced for confirmation. Sequences from both forward and reverse strands were assembled, and manually edited using Sequencher version 4.8 (Gene Codes Corporations). Based on BLAST analysis of the GenBank database (NCBI), each allele was assigned a number [10]. GenBank accession numbers with corresponding allele numbers are listed in the supplementary information (Table S2). For the microsatellite analysis two software packages were used: Magellan, a freely available software package developed by Dee Carter's laboratory at the University of Sydney (http://www.medfac.usyd.edu.au/people/academics/profiles/dcarter.php) [38], and the Tandem Repeat Finder (TRF) software package developed at Boston University [39]. Sequences were assembled and edited using Sequencher version 4.8 (Gene Codes Corporations) and aligned using the Clustal X version 2.0 software package [40].

### Fertility Analysis

Mating analysis was conducted on Murashige and Skoog (MS) media, which contains myo-inositol that stimulates mating [41]. Isolates were incubated at room temperature (24°C) in the dark for 10 to 14 days under dry conditions. Fertility was assessed by microscopic examination for hyphae, fused clamp cells, basidia, and basidiospore formation.

### Virulence

Infection assays in the heterologous host *Galleria mellonella* (greater wax moth) were conducted with a method similar to previous studies [42]. All animals were purchased in bulk and a single shipment used for each replicate virulence test (Van der Horst Wholesale, St. Marys, Ohio). The infectious inoculum was injected directly into the hemolymph via the penultimate or ultimate pseudo-pods using a Hamilton syringe (Hamilton USA). All experiments were conducted in duplicate with an infectious dose of 1.0×10^5^ cells, and larvae were maintained in a 37°C controlled environment. For each replicate virulence assay, 12–19 larvae were infected for each strain analyzed. Animals were monitored at 24-hour intervals, and mortality was defined as the cessation of movement upon probing and development of a distinctive dark colorization.

## Results

### Clinical presentation of an unusual *Cryptococcus* infection

**Figure 1:**
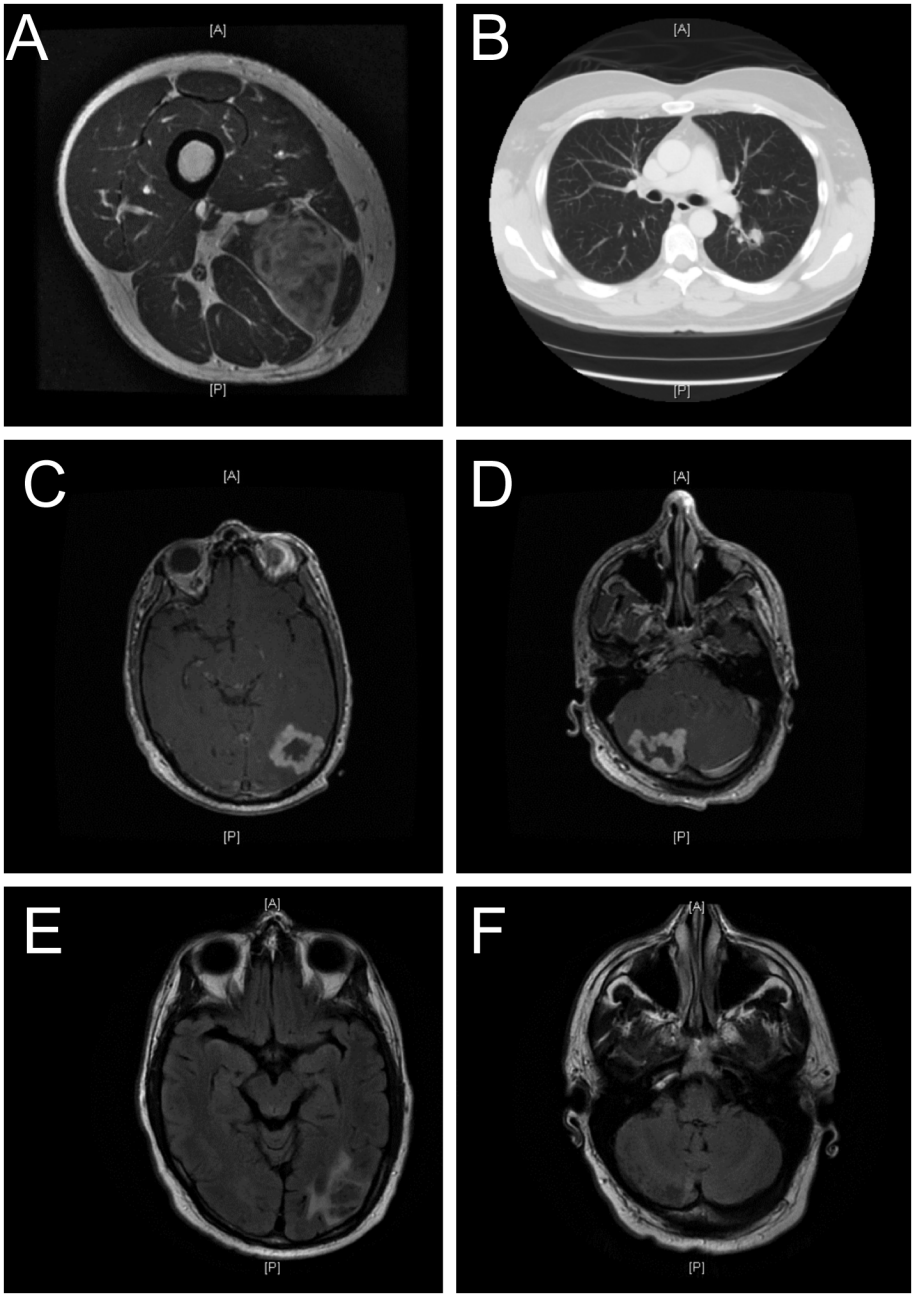
Imaging from diagnosis through recovery depicting the clinical course of *C. gattii* infection. A) MRI imaging of the upper thigh cryptococcoma. B) CT imaging of a pulmonary nodule, likely to be a cryptococcal granuloma. C–D) MRI imaging of brain cryptococcomas after seizure presentation at the emergency room. E–F) MRI imaging of brain cryptococcomas after long-term fluconazole treatment, with reduced mass.

The patient is a 46-year-old male with an unremarkable past medical history who noticed a hard mass on his medial right thigh. The mass enlarged over the course of three weeks, but was not painful, and the patient had no other symptoms. His primary care physician evaluated the mass with a magnetic resonance imaging (MRI) scan, which showed a 5×4×4 centimeter mass in the inner mid right thigh involving the adductor magnus muscle. The mass had mild heterogeneous enhancement on T2 imaging (Figure 1A) and some changes consistent with limited surrounding edema. The radiographic appearance was most consistent with a malignancy, and he underwent further radiographic investigation with chest, abdominal, and pelvic computed tomography (CT) scans.

The chest CT scan revealed a spiculated 1.6×2 centimeter left lower lobe lung mass with multiple, bilateral sub-centimeter pulmonary nodules (Figure 1B). The patient underwent a percutaneous biopsy of the thigh mass, and pathology demonstrated soft tissue necrosis and thick walled, encapsulated organisms that stained with Gomori Methenamine Silver (GMS) and mucicarmine. The entire mass was surgically resected, and cultures from the mass grew *Cryptococcus*. Further evaluation demonstrated a serum cryptococcal antigen of 1∶32, negative HIV serologies, and a normal CD4 lymphocyte count.

No other symptoms were revealed by a detailed medical history, but the patient was regularly exposed to birds in the workplace. However, extensive local sampling of the workplace environment and birdcages failed to reveal a local source of infection. The patient was placed on 400 mg fluconazole a day, and did well until three months later when he presented with a tonic-clonic seizure. An MRI scan showed a large, enhancing mass in the left parietal lobe and a similar appearing mass in the right cerebellar hemisphere (Figure 1C–D). A biopsy of the parietal mass showed numerous yeast cells, and cultures grew *Cryptococcus*.

After diagnosis, the patient was initially treated with amphotericin B and flucytosine, but due to renal toxicity/nephropathy was changed to oral high-dose fluconazole (800 mg daily). The large size of the mass lesions despite antifungal therapy prompted consideration that the infection might be due to *Cryptococcus gattii*, and serotyping with commercial monoclonal antibodies (Iatron, Tokyo, Japan) revealed the isolate to be serotype B, consistent with *C. gattii* (T. Mitchell, A. Litvintseva, personal communication). The patient had traveled to San Francisco five months prior to his original presentation. He had never been to the Pacific Northwest, Australia, South America or other *C. gattii* endemic regions and his only foreign travel had been to Western Europe.

Sixteen months after the seizure he was completely asymptomatic on fluconazole 400 mg a day, and showed a significant decrease in the cerebral cryptococcomas upon reevaluation with magnetic resonance imaging (Figure 1E–F). The patient is currently asymptomatic and continuing fluconazole treatment as of February 2009 with a cryptococcal antigen titer testing positive at 1∶4. The patient will continue treatment, and be re-scanned in the spring of 2009.

### Phenotypic analysis reveals isolates are *C. gattii*

**Figure 2:**
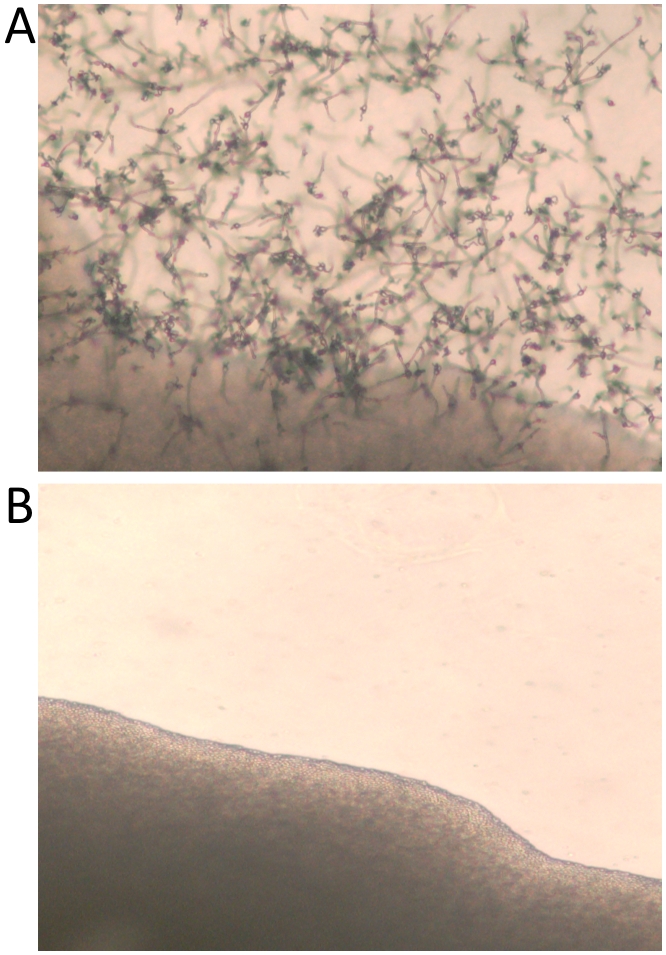
Clinical isolates exhibit mating differences. All mating cultures were incubated at room temperature in the dark, for 10 to 14 days in dry conditions using the mating type a tester isolate B4546 as a partner on Mirashige and Skoog Media. The brain biopsy isolate exhibits reduced fertility as evidenced by a marked delay and paucity in hyphal growth (A) whereas hyphal growth, basidia, and basidiospore formation indicative of sexual reproduction is evident in matings with the leg biopsy isolate (B). All mating experiments were repeated and representative images are shown here.

Initial identification and confirmation that the leg (EJB1-L) and brain (EJB2-B) isolates were *C. gattii* was completed using several phenotypic and molecular tests. Isolates were confirmed to be *C. gattii* based on melanin production on Staib niger seed agar, production of urease on Christensen's agar, and resistance to canavanine with glycine utilization on CGB agar (Figure S1). Capsule production was assayed through India ink exclusion of cells grown at 37°C for 72 hours in DMEM, resulting in high levels of polysaccharide capsule indicative of pathogenic *Cryptococcus* species (Figure S1). Isolates were determined to be mating type α based on controlled mating assays with *C. gattii* VGIII isolates NIH312α and B4546**a**. Although both isolates ultimately were able to complete the sexual cycle with the **a** mating tester, and are therefore α mating type, there was a severe and reproducible defect in hyphae and basidiospore formation in mating assays with isolate EJB2-B when compared with EJB1-L (Figure 2), with similar results obtained using a mating type **a** *crg1* tester strain (data not shown). Differences in mating ability were also observed in other similar or identical genotype isolates (B4496 and E296 (high fertility) vs. PAT12ISO1 and E310 (low fertility)) (Figure S2). There were no other significant differences between the isolates when comparing melanin production, growth at 37°C, growth on CGB agar, and urease and capsule production (Figure S1).

### Molecular analysis reveals isolates are VGI molecular type

**Figure 3:**
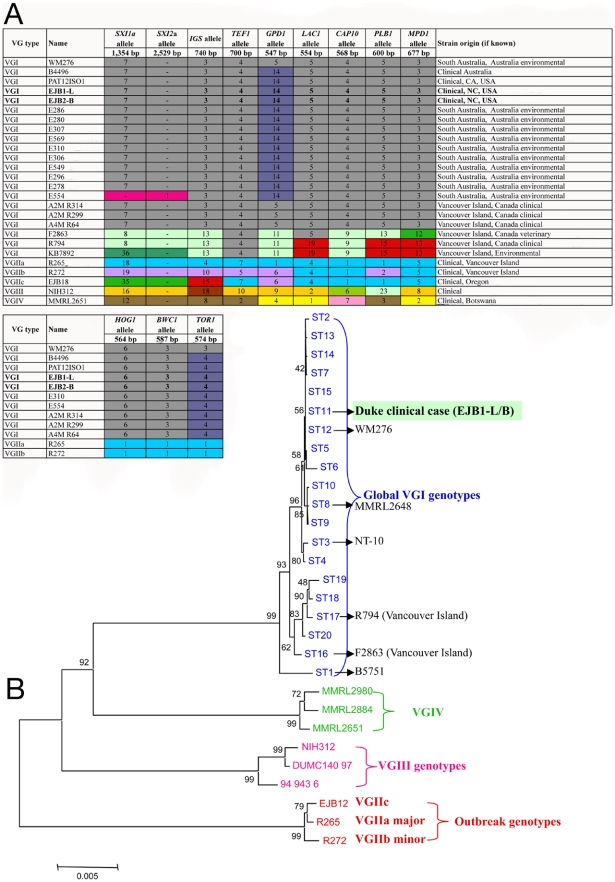
Molecular typing and phylogeny of the North Carolina clinical isolates with global isolates. A) MLST reveals the leg and brain isolates are identical with each other, and also identical with a distinct genotype predominantly from Australia, and in a single clinical case from California, USA. B) Neighbor joining phylogenetic analysis based on sequences from seven MLST loci in panel A (*SXI* idiomorphs not included) illustrates discrimination between four molecular types (VGI-VGIV), and displays the relationship of the clinical case presented in this study to global genotypes observed, including all VGI genotypes thus far reported (including the Vancouver Island VGI genotypes), and the Vancouver Island/Pacific NW outbreak genotypes VGIIa/major, VGIIb/minor, and VGIIc. Note that not all sequence types and strains in (B) are represented in (A).

Molecular studies were conducted to determine if this clinical case was in any way related to the outbreak of *C. gattii* on Vancouver Island that has now expanded to mainland British Columbia, Washington, and Oregon. Genomic DNA of each isolate was analyzed by MLST at a minimum of eight loci (Figure 3). In addition to the mating analysis to determine the mating type of each isolate, PCR and sequence analysis were used to detect either of the two mating type-specific idiomorphs of the *SXI* genes. Amplification and sequence analysis of the sex specific *SXI1*α mating type gene, as well as the absence of the mating type specific *SXI2* **a** gene, confirmed that both isolates had the α mating type allele (*MAT*α). MLST analysis also showed that all MLST alleles were identical between both the leg biopsy (EJB1-L) and brain biopsy (EJB2-B) isolates, and each typed as *C. gattii* molecular type VGI (Figure 3).

Detailed analysis of the molecular profile, and comparison with >280 global isolates including 85 VGI isolates indicated that while the isolates were highly similar to the most common molecular type (VGI), they were identical to only a unique subset in the collection. To examine if these isolates were at all related to the ongoing *C gattii* outbreak on Vancouver Island, Canada, and the Pacific NW, we examined all VGI isolates from this region in detail. A total of six VGI isolates have been reported from Vancouver Island, and none of these matched the NC clinical case based on MLST analysis [8]. Three Vancouver Island isolates (F2863, R794, and KB7892) are quite diverged from the NC case (EJB1-L and EJB2-B) based on MLST analysis (Figure 3). Three others (A2M R314, A2M R299, and A4M R64) share 7 MLST alleles but differ at the GPD1 allele (Figure 3). Thus, none of the Vancouver Island isolates are a direct match to the NC clinical case isolates. Of the six reported Vancouver Island VGI isolates, there are four different MLST genotypes (Figure 3A). Of these genotypes two are clinical (human), one veterinary, and one environmental. There are no identical matches between the Vancouver Island VGI environmental and clinical isolates. Thus, there are VGI genotypes in the environment but infections may have been acquired elsewhere, although wild animal cases are most often locally acquired. Currently, no VGI cases have been reported in Washington or Oregon states, and the incidence of VGI on Vancouver Island and surrounding areas in the Pacific NW region remains low.

Of the eleven VGI molecular type isolates with an identical MLST genetic profile to the NC isolate, nine were from the environment in Renmark, Australia, and were isolated from *Eucalyptus* tree hollows (Figure 3A) [43]. An additional Australian environmental isolate (E554) shared all loci with the NC clinical case with the exception of the mating type specific *SXI* idiomorph, because this isolate is mating type **a** (Figure 3A). The two MLST matched clinical isolates were both from human infections, one from Australia and one from a blood sample from a liver transplant recipient treated in San Francisco, California (Figure 3) [32]. To further discriminate isolates, the MLST analysis was extended to include three additional loci. This analysis included the NC, CA, Vancouver Island, and Australian clinical cases as well as representative Australian environmental isolates. However, no polymorphisms were detected in the three clinical isolates harboring the NC clinical case genotype using the additional coding markers (Figure 3A). Based on neighbor joining phylogenetic analysis [40], in which seven MLST loci were concatenated into 5.7 kb of contiguous sequence, the four discrete VG molecular types were clearly distinct, and the VGI group was separated into 20 sequence types (ST) (Figure 3B). All isolates with an identical MLST type to the NC clinical case grouped into sequence type 11 (ST-11), and were closely related to the more common sequence type 12 (ST-12), which harbors the VGI molecular type genomic reference strain WM276.

**Figure 4:**
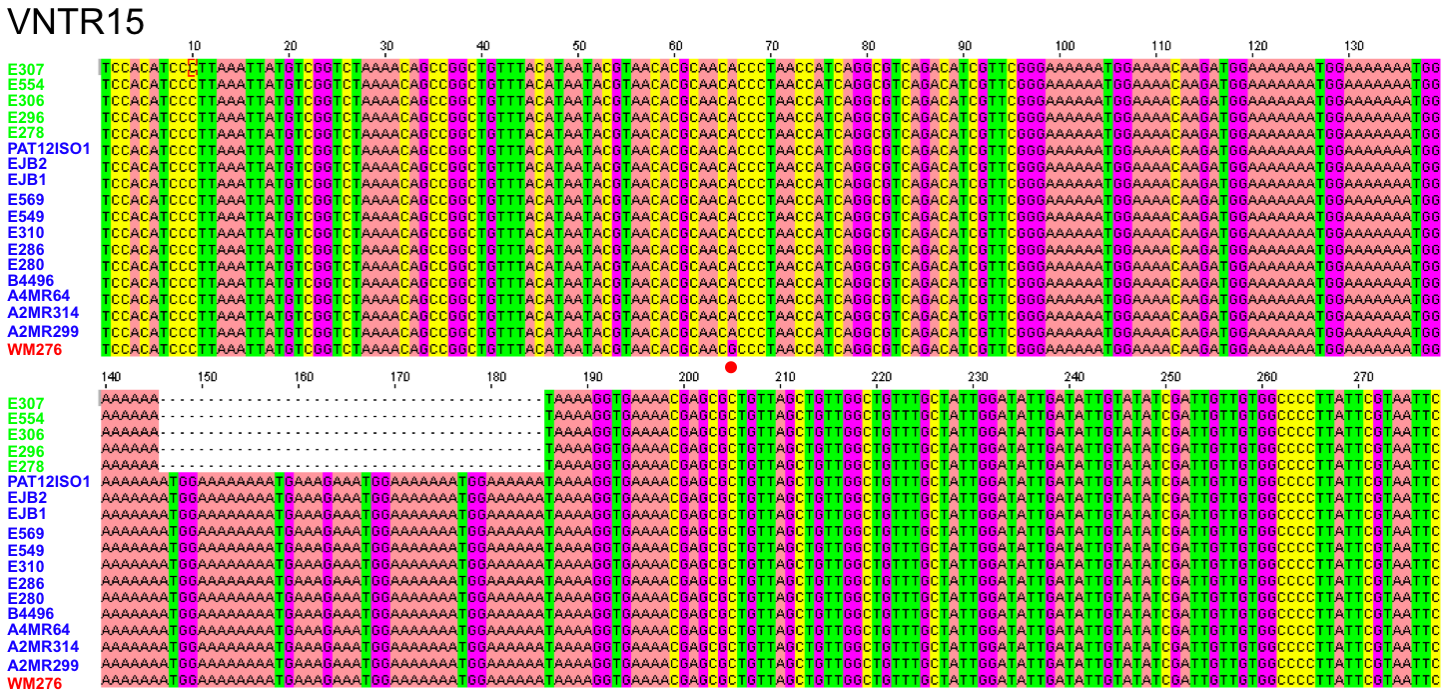
DNA sequence alignment of VNTR15 among 18 VGI isolates of *C. gattii*. The noncoding VNTR marker divides the isolates with an identical MLST profile into two groups: one group contains two NC clinical isolates which are identical to one another, the other clinical isolates, and five of the ten Renmark isolates, and the second group includes the remaining five Renmark isolates. A 40 bp deletion was observed in the following isolates: E307, E554, E360, E296, and E278. In addition, a single nucleotide polymorphism (SNP) (66A → 66G) discriminated the VGI type strain isolate WM276 from the other 17 VGI isolates (see red circle).

**Figure 5:**
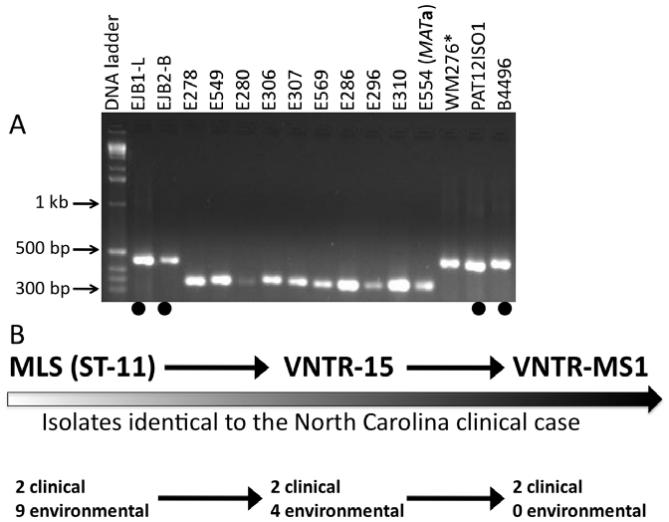
Molecular typing reveals that the Australian environmental isolates are distinct from clinical isolates. A) PCR product size differences at locus VNTR-MS1 reveals that environmental isolates from Renmark harbor a deletion compared to the clinical isolates with the same MLST genotype. Agarose gel electrophoresis of the VNTR-MS1 marker, illustrating an ∼100 bp difference between the larger (EJB1-L, EJB2-B, PAT12ISO1, B4496, WM276*), and smaller (E278, E549, E280, E306, E307, E569, E286, E296, E310, E554) PCR products. * Denotes that the sequenced type strain WM276 is not identical to the other clinical isolates. The bold circles represent four clinical isolates (from three cases) that type as identical through all genotypic tests conducted. B) A linear representation of the progressive genotypic analysis. As the number of markers increased, along with the genetic variability, the number of isolates typing as identical to the North Carolina clinical case decreased. At the end of the genotypic studies only two clinical isolates, previously identified from Australia and California, typed as identical with the present NC clinical case.

Multilocus sequence typing is a powerful approach to discriminate isolates in a sequence-based method, but also relies almost exclusively on the genomic coding sequences of conserved genes. For the analysis of highly clonal populations such as the VGI molecular type, variable non-coding and intergenic sequences, particularly tandem repeats, can enable more detailed characterizations of populations by allowing closely related isolates to be distinguished. Using microsatellite markers developed with the Magellan Software suite, and Variable Number of Tandem Repeat (VNTR) analysis with the Tandem Repeat Finder (TRF) software, strains harboring identical MLST profiles to the isolates from the patient in this study were analyzed (Figure 3). Analysis of three independent VNTR markers showed that two of the markers were identical in all ST-11 isolates (Figure S3), while marker VNTR-15 was able to discriminate five of the Australian environmental isolates from all clinical isolates (NC-clinical, CA-clinical, Australia-Clinical) and the other five environmental isolates from Australia with this molecular genotype, due to a 40 base pair deletion within the tandem repeat region (Figure 4). In addition, analysis of microsatellite marker MS1 clearly distinguished the Renmark environmental isolates, which produced a smaller PCR product (∼380 bp) than the three clinical isolates and VGI control isolate WM276 (∼480 bp) (Figure 5A). A high GA content and repetitive nature of this locus impeded sequence analysis, as polymerase slippage resulted in low-quality sequence results. However, partial sequence affirmed that the products were specific (data not shown). Isolate WM276 was distinguished from the three clinical isolates based on a SNP in the *GPD1* MLST allele locus, a 4 bp deletion in the *TOR1* MLST allele, a SNP in VNTR3, and a SNP in VNTR15. In addition, the VNTR3 analysis further differentiated the NC, SF, and Australia ST-11 clinical isolates of interest from the Vancouver Island VGI ST-12 isolate A4MR64 (Figure S3). Overall, the analysis of variable sequences further discriminated the three clinical isolates with this unique MLST type from the environmental isolates from Renmark, Australia with the same 8-loci MLST genotype (Figure 5B). These data indicate that while MLST is informative, it lacks sufficient resolution to discriminate isolates with highly similar albeit distinguishable genotypes.

### *C. gattii* VGI isolates are virulent in a heterologous host

**Figure 6:**
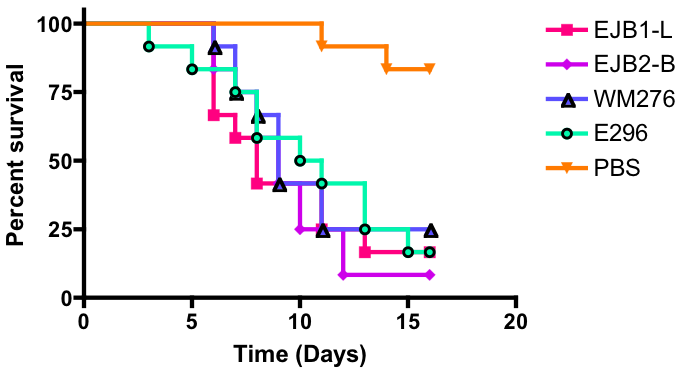
North Carolina clinical isolates are pathogenic in a heterologous host. Groups of 12 to 19 larvae of *Galleria mellonella* were each infected with an infectious inoculum of 1.0×10^5^ cells of isolates EJB1-L, EJB2-B, WM276, and E296. Survival was monitored and plotted daily for 16 days. All isolates were significantly virulent (p<0.005) in comparison with the mock control (sterile PBS) infection. The experiment was replicated in duplicate with similar results in each replicate, and representative results are shown here.

To determine the relative virulence between environmental and clinical isolates of the same MLST type as the North Carolina clinical case, infection assays were conducted in the heterologous host, *Galleria mellonella* (greater wax moth). The use of non-mammalian hosts to study fungal virulence and host defense has been increasingly applied and is now well established [42], [44], [45]. Although Lepidoptera are distantly related to mammals, results in the wax moth larval model have been highly correlated with results using murine infection models [42], [46], [47]. In the virulence assay, isolates were injected at an infectious inoculum of 1×10^5^ cells, using 12 to 19 larvae per strain per replicate at 37°C. The selected isolates analyzed were all significantly pathogenic compared to PBS control infections (p<0.005) (Figure 6). There was no marked difference between ST-11 environmental and clinical isolates (p>0.2). In addition, the VGI type strain control isolate (WM276) showed no difference in virulence to the tested isolates (p>0.2). This uniformity in virulence between environmental and clinical isolates is consistent with direct acquisition of infections from an environmental source.

## Discussion

Our findings document the first *C. gattii* case reported in the Eastern region of the United States. The most parsimonious explanation is that an otherwise healthy patient was exposed in California, resulting in disease presentation in North Carolina approximately 4 to 6 months after travel to an endemic region. This case highlights an overall increase in *C. gattii* infections in apparently immunocompetent individuals in temperate climates. The patient had no recent travel history to the Pacific Northwest or to other *C. gattii* endemic regions, and based on molecular typing the infectious isolate is not related to any of the VGII or VGI isolates causing the ongoing Vancouver Island outbreak, and its emergence into mainland British Columbia, Washington, and Oregon [18], [20], [22], [23], [48]. These VGI clinical isolates are also distinct from molecular types VGIII and VGIV (Figure 3B). *C. gattii* molecular type VGI has never been reported from the environment in California, but it seems likely to be present there based on successful environmental isolation of *C. gattii* molecular types VGII and VGIII in regions of California, including San Francisco and San Diego, and the abundant presence of the host *Eucalyptus* trees throughout the state [34], [49].

**Figure 7:**
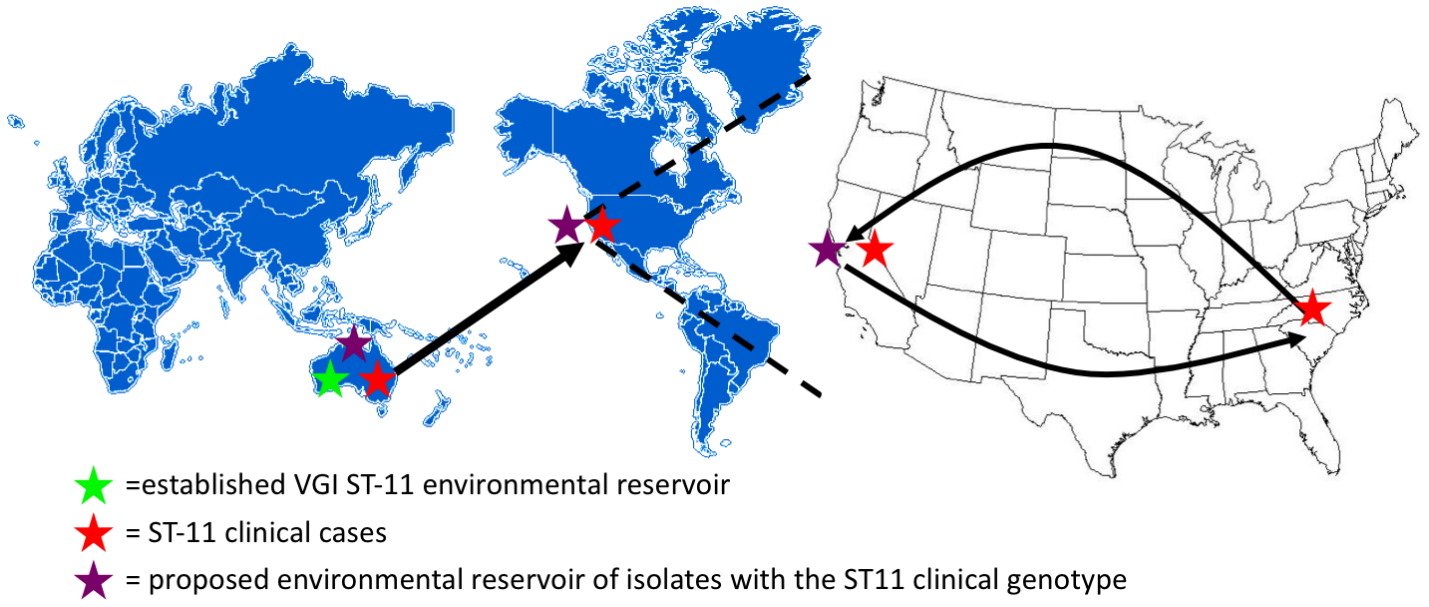
Proposed model for the emergence of *C. gattii* in the United States. The original source is postulated to be Australia, where identical clinical and closely related environmental isolates have been reported. Given that human-human transmission other than introgenic is unknown, the most parsimonious model for geographically dispersed clinical isolates is that isolates identical to the clinical cases are present in the environments in Australia and California, USA. In this model, the patient from the Southeastern United States traveled to the endemic area of CA, was exposed to the pathogen by inhalation, and ultimately returned to present with disseminated disease in North Carolina, USA.

Our current model suggests that this unique MLST VGI genotype is located in the environment and clinical setting in Australia, with probable environmental colonization in California, resulting in human infections (Figure 7). These conclusions are based on broad MLST analysis as well as analysis of four hyper-variable tandem repeats located throughout the *C. gattii* genome. The VNTR analysis showed that environmental isolates all differ at one or two rapidly evolving genomic regions, but clinical and environmental isolates that are identical at eight MLST loci are quite closely related. All environmental isolates from Australia were collected from a single location, and slight diversity in geographic regions is expected [50]. In October 2007, seventy samples were collected from *Eucalyptus* trees in San Francisco. No isolates of *C. neoformans* or *C. gattii* were obtained. Therefore, expanded sampling will be necessary to definitively elucidate the environmental source of the clinical isolates. We therefore posit that the patient in this report traveled to the San Francisco region, and was there exposed to infectious spores or desiccated yeasts, resulting in colonization of the lungs, and dissemination, disease progression, and presentation with systemic infection of the leg and brain (Figure 7). Each of the three clinical cases caused by this VGI genotype occurred independently as there is no evidence of direct human-to-human transmission with this pathogen, other then rare introgenic transmissions. In addition, if in vivo changes during infection were responsible for the clinical and environmental isolate molecular differences we would have expected the clinical isolates to differ from each other, and this is not the case (Figure 5B). Therefore, the most parsimonious explanation is independent environmental-to-human exposure, with the exact source yet to be identified in Australia and in California. These studies demonstrate that increased typing resolution can be achieved by combining coding and noncoding genomic markers, which are abundantly available for fungal pathogens with publicly available genome sequences.

Other clinical cases of *C. gattii* due to travel in endemic areas have been recently reported. In 2007, a 51-year-old HIV negative male from Denmark presented with *C. gattii* disease in Europe after traveling to Vancouver Island, Canada [37]. Molecular analysis established that the isolate was identical to the VGIIa/major genotype responsible for the Vancouver Island outbreak [37]. Additionally, a study from the British Columbia Centre for Disease Control indicated that intra-island travel or tourism on Vancouver Island to highly endemic areas increases the likelihood of contracting cryptococcosis [36]. These studies demonstrate the importance of human travel to endemic areas as it relates to the acquisition and presentation of cryptococcal disease. Many clinical laboratories in non-endemic regions do not differentiate *C. neoformans* infections from *C. gattii* infections; therefore, the incidence of travel associated *C. gattii* infections may be more common than currently appreciated.

While many of the studies related to *C. gattii* infections in the United States and Canada have focused on the Vancouver Island outbreak, it has been known for several decades that *C. gattii* is also endemic to the environment in regions of California, causing infections in both animals and humans. In 1991, an isolate of *C. gattii* VGIIa/major outbreak genotype was sampled from a *Eucalyptus camaldulensis* tree at Fort Point Park, in San Francisco, indicating a natural occurrence in the same host tree that *C. gattii* is most commonly associated with in Australia [10], [34]. Several studies have also documented a likely endemic zone in Southern California. A large cohort of HIV positive patients with cryptococcal infections from Los Angeles hospitals revealed that the infections were principally the result of *C. gattii* serotype C infections [31]. In addition, *C. gattii* VGI has been isolated from a marine mammal case (bottlenose dolphin) near San Diego, and *C. gattii* VGIII has been isolated from the natural environment in the San Diego, California area [33], [51]. These studies provide strong evidence that multiple molecular types (VGI, VGII, VGIII) have been reported in California indicative of a likely reservoir, possibly in *Eucalyptus* and other tree species.

Hypotheses regarding the spread of global fungal pathogens from common associations with plants have been previously reported. It was recently shown that a basidiomycete, the sugarcane fungal pathogen *Ustilago scitaminea,* was dispersed from Asia to America and Africa due to its association with infected plant material [52]. *C. gattii* has been isolated from *E. camaldulensis* trees in San Francisco, a common host tree species, and *C. gattii* was recently found to complete its natural sexual cycle during co-culture with this plant under laboratory conditions [34], [41], [53]. Population genetics studies of individual trees of *E. camaldulensis* have also shown that both same-sex and opposite-sex mating is likely occurring in this environmental niche [54]. These reports demonstrate the likelihood that this unique VGI genotype migrated due to plant association, and also leaves open the possibility of sexual reproduction occurring in natural populations of this genotype in the environment.

Sexual reproduction among closely related genotypes has the potential to increase pathogenicity of fungi and parasites [55], [56]. In *C. gattii* it has been postulated that same-sex mating among closely related isolates could have both contributed to the formation of the hyper-virulent VGIIa/major genotype responsible for the Vancouver Island outbreak and to the ongoing production of infectious propagules [10], [57], [58]. Another well-studied system is the eukaryotic parasite *Toxoplasma gondii,* where limited mating among clonal lineages is hypothesized to have resulted in increased virulence, and may also have enabled this pathogenic microbe to be orally transmitted from animals to humans [59], [60]. The reasons for mating differences among the isolates from the leg and brain of the North Carolina *C. gattii* case may be genetic, or epigenetic, as all other analysis indicates that these isolates are from a single rather than mixed infection. Although the exact roles of *C. gattii* sexual reproduction as it relates to virulence remain to be elucidated, the ability of virulent isolates to retain mating ability suggest this process may play a significant role in virulence and possibly also in the production of infectious propagules.

As *C. gattii* continues to emerge in the United States and elsewhere it is clear that the standardization of clinical testing to identify species (*C. neoformans/C. gattii*) and cryptic species (VGI/VGII/VGIII/VGIV) is essential. As illustrated by this case, *C. gattii* infections may be particularly complicated to treat due to their predilection to form cryptococcomas, especially in the brain, which are difficult to manage and risk to surgical resection is considerable. Although clinical laboratory testing at Duke University confirmed that the leg and brain biopsy isolates were both fluconazole sensitive, because of disease progression and the occurrence of cryptococcomas, in which drug access is limited, clinical management was difficult. Although the overall incidence of *C. gattii* disease in the United States remains low, an increasing number of cases in California, Oregon, Washington, and now North Carolina among immunocompetent and immunocompromised individuals raise the possibility that these represent the onset of an emergence in the temperate climate of the Western United States.

## Supporting Information

Figure S1 Clinical and Australian environmental *C. gattii* VGI isolates exhibit mating differences. All mating cultures were incubated at room temperature in the dark for 14 days in dry conditions using the mating type a tester isolate B4546 as a partner on Mirashige and Skoog Media. The clinical isolate B4496 (top left) and the environmental isolate E296 (top right) are fertile. There is a marked delay and paucity, or no hyphal growth in matings with the clinical isolate PAT12ISO1 (bottom left), and the environmental isolate E310 (bottom right).
(51.87 MB TIF)

Figure S2 Phenotypic characterization of *C. gattii* isolates. In each panels A and B control isolates are as follows: top left, *C. albicans* control (CA, isolate SC5314) Isolate; bottom left, *C. neoformans* control (CN, isolate H99); bottom right, *C. gattii* control (CG, isolate R265). In panel A, the experimental isolate (upper right) is EJB1-L. In panel B, the experimental isolate is EJB2-B. Each isolate of *C. gattii* produced melanin on Staib niger seed agar (brown pigmentation), produced urease on Christensen's agar (pink coloration), and was resistant to canavanine and utilized glycine on CGB agar (growth and blue coloration). C) Isolates EJB1-L (left panel), and EJB2-B (right panel) each show similar capsule sizes when exposed to India ink following growth on DMEM media for 48 hours at 37Â°C.
(5.82 MB TIF)

Figure S3 Analysis of VNTR markers. DNA sequence alignment of VNTR markers among 18 VGI isolates of *C. gattii*. The VNTR3 marker discriminates two of the ST12 isolates (WM276 and A4MR64) from the other 16 VGI isolates. VNTR7 shows 100% sequence identity between all 18 isolates examined (red circle indicates SNP).
(38.91 MB TIF)

Table S1 (0.05 MB DOC)

Table S2 (0.06 MB DOC)
