# Supplementary figures and images for: First Reported Case of Cryptococcus gattii in the Southeastern USA: Implications for Travel-Associated Acquisition of an Emerging Pathogen
Source: PLoS One. 2009 Jun 10;4(6):e5851. doi: 10.1371/journal.pone.0005851 (PMC2689935; doi:10.1371/journal.pone.0005851)

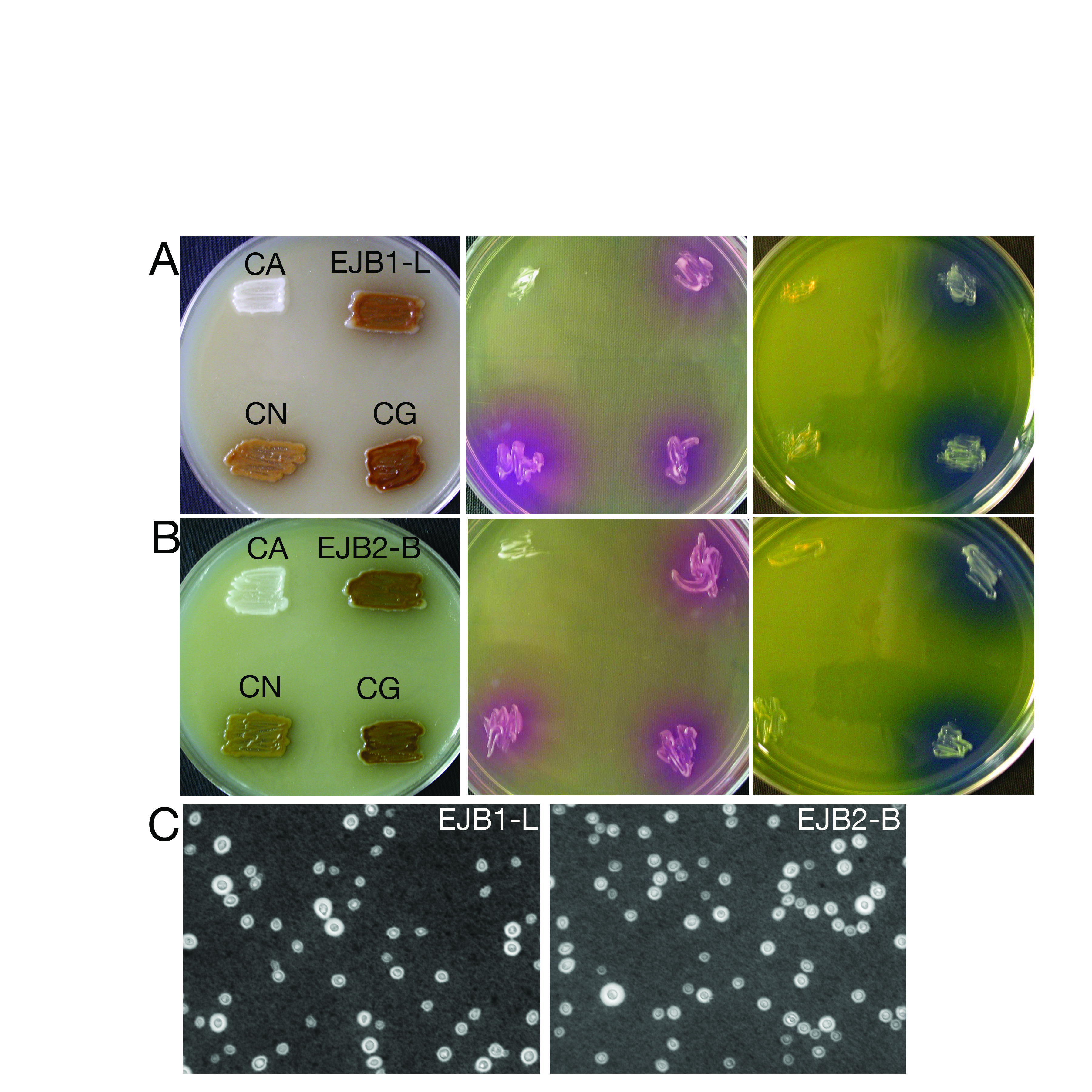

Supplement: Figure S1 — Clinical and Australian environmental C. gattii VGI isolates exhibit mating differences. All mating cultures were incubated at room temperature in the dark for 14 days in dry conditions using the mating type a tester isolate B4546 as a partner on Mirashige and Skoog Media. The clinical isolate B4496 (top left) and the environmental isolate E296 (top right) are fertile. There is a marked delay and paucity, or no hyphal growth in matings with the clinical isolate PAT12ISO1 (bottom left), and the environmental isolate E310 (bottom right). (51.87 MB TIF) [file pone.0005851.s001.tif]

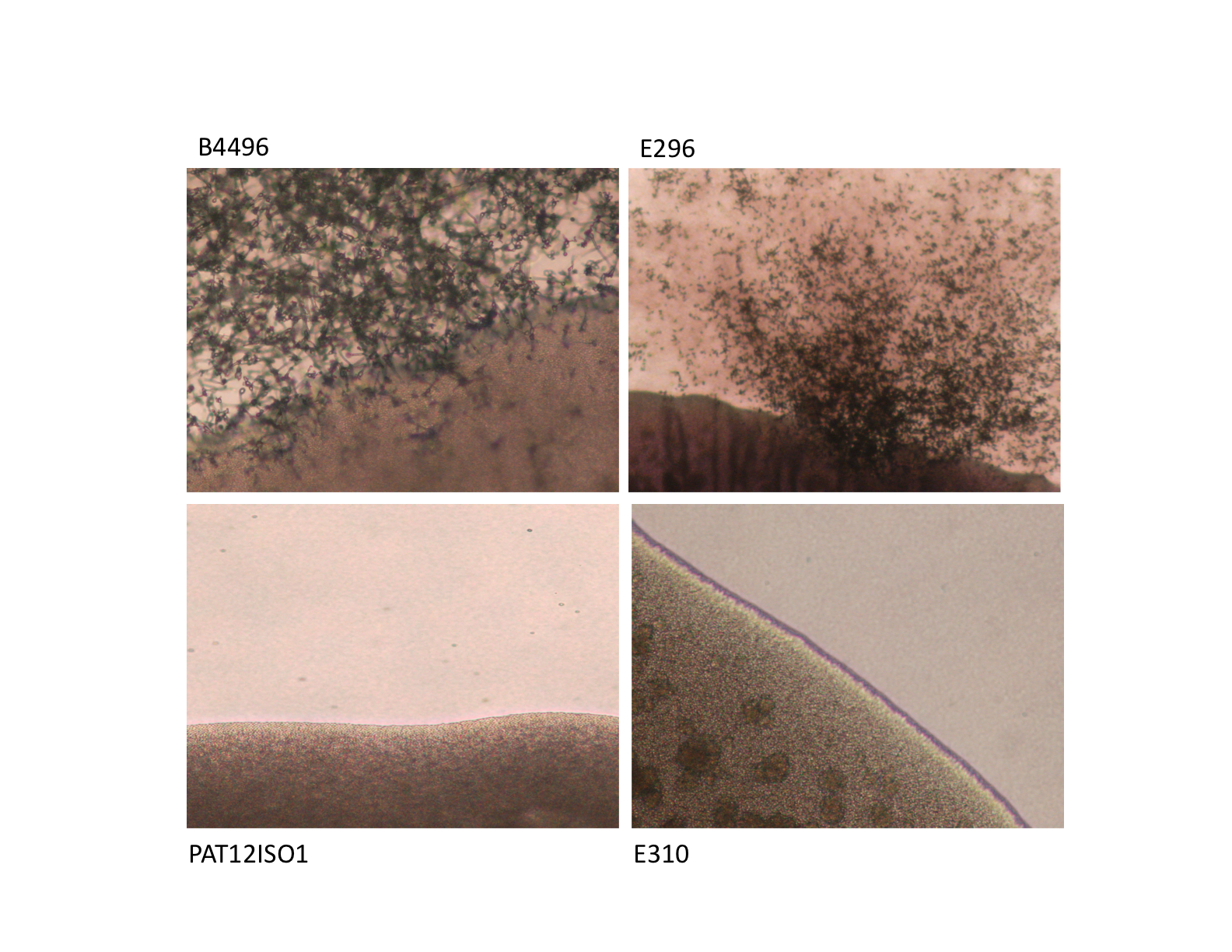

Supplement: Figure S2 — Phenotypic characterization of C. gattii isolates. In each panels A and B control isolates are as follows: top left, C. albicans control (CA, isolate SC5314) Isolate; bottom left, C. neoformans control (CN, isolate H99); bottom right, C. gattii control (CG, isolate R265). In panel A, the experimental isolate (upper right) is EJB1-L. In panel B, the experimental isolate is EJB2-B. Each isolate of C. gattii produced melanin on Staib niger seed agar (brown pigmentation), produced urease on Christensen's agar (pink coloration), and was resistant to canavanine and utilized glycine on CGB agar (growth and blue coloration). C) Isolates EJB1-L (left panel), and EJB2-B (right panel) each show similar capsule sizes when exposed to India ink following growth on DMEM media for 48 hours at 37Â°C. (5.82 MB TIF) [file pone.0005851.s002.tif]

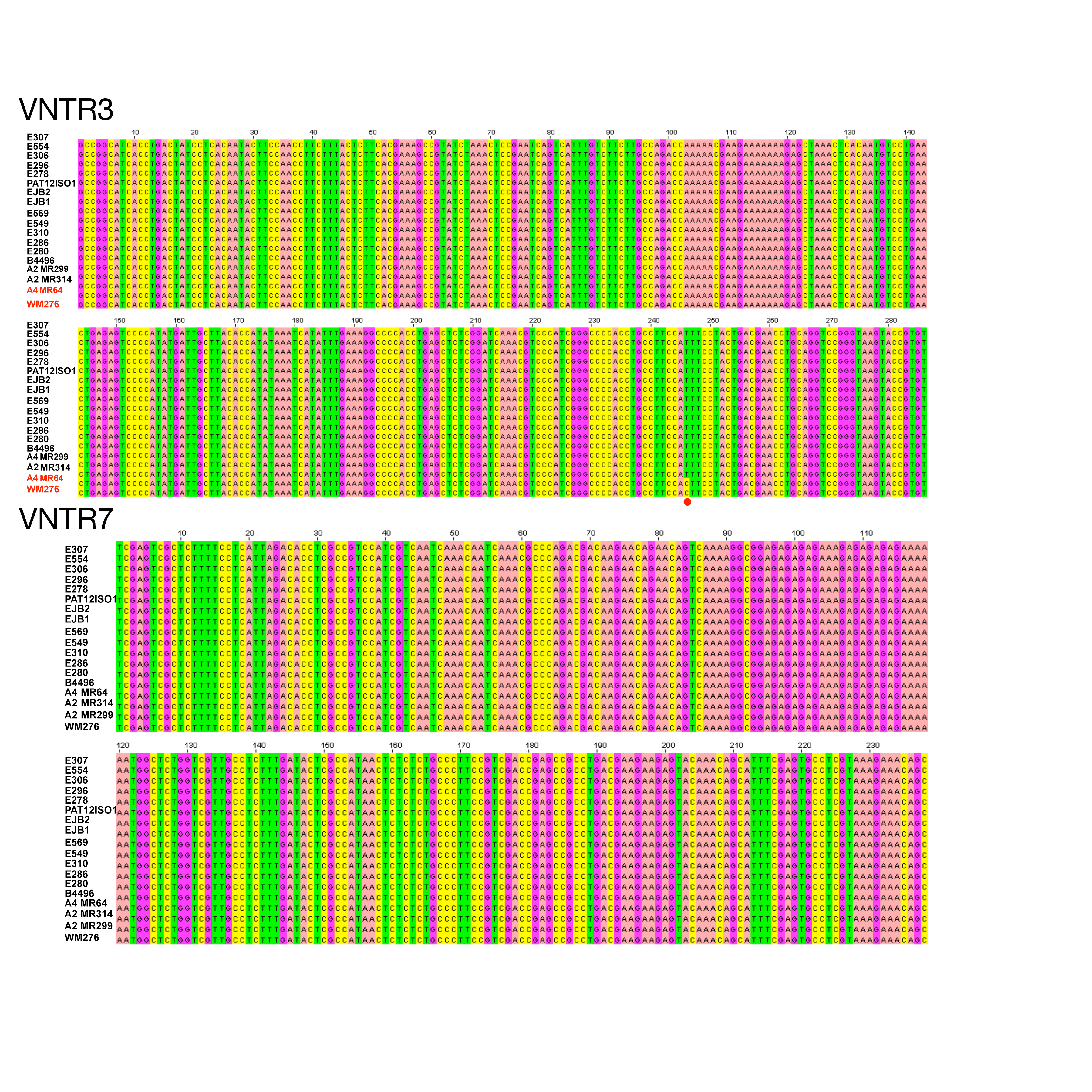

Supplement: Figure S3 — Analysis of VNTR markers. DNA sequence alignment of VNTR markers among 18 VGI isolates of C. gattii. The VNTR3 marker discriminates two of the ST12 isolates (WM276 and A4MR64) from the other 16 VGI isolates. VNTR7 shows 100% sequence identity between all 18 isolates examined (red circle indicates SNP). (38.91 MB TIF) [file pone.0005851.s003.tif]
