# Supplementary material for: First Reported Case of Cryptococcus gattii in the Southeastern USA: Implications for Travel-Associated Acquisition of an Emerging Pathogen
Source: PLoS One. 2009 Jun 10;4(6):e5851. doi: 10.1371/journal.pone.0005851 (PMC2689935; doi:10.1371/journal.pone.0005851)
Supplement: Table S1 — (0.05 MB DOC) [file pone.0005851.s004.doc]

Table S1. Oligonucleotides used in this study.

| Name | Description | Oligonucleotide Sequence |
| --- | --- | --- |
| JOHE10451 | *SXI1*α 5’ MLST primer | TACATCACCGGTCATATCTGC |
| JOHE10452 | *SXI1*α 3’ MLST primer | CTGGAGAAGCGCCTCACTGGA |
| JOHE14115 | *SXI1*α alternative 5’ MLST primer | AGGGTACGTTTGAGGCCAGTT |
| JOHE14116 | *SXI1*α alternative 3’ MLST primer | GAAAGCGTTGGCAAGGAATGA |
| JOHE10453 | *SXI2***a** 5’ MLST primer | TGATCGCACGAGCCAAATCCC |
| JOHE10454 | *SXI2***a** 3’ MLST primer | GGCTTCCTGACAACACTTCTA |
| JOHE14408 | *IGS* 5’ MLST primer | ATCCTTTGCAGACGACTTGA |
| JOHE14409 | *IGS* 3’ MLST primer | GTGATCAGTGCATTGCATGA |
| JOHE14976 | *TEF1* 5’ MLST primer | GCACGCTCTTCTCGCCTTCAC |
| JOHE14977 | *TEF1* 3’ MLST primer | GTAGTCGGCGTAGGTCTCAAC |
| JOHE14968 | *GPD1* 5’ MLST primer | CCACCGAACCCTTCTAGGATA |
| JOHE14969 | *GPD1* 3’ MLST primer | CTTCTTGGCACCTCCCTTGAG |
| JOHE14970 | *LAC1* 5’ MLST primer | AACATGTTCCCTGGGCCTGTG |
| JOHE14971 | *LAC1* 3’ MLST primer | ATGAGAATTGAATCGCCTTGT |
| JOHE14386 | *CAP10* 5’ MLST primer | CCGGAACTGACCACTTCATC |
| JOHE14387 | *CAP10* 3’ MLST primer | GCCCACTCAAGACACAACCT |
| JOHE14974 | *PLB1* 5’ MLST primer | CTCTCATTGTTCGCCGCTACT |
| JOHE14975 | *PLB1* 3’ MLST primer | GGAAGCCGAGGTCTGATTTGG |
| JOHE14972 | *MPD1* 5’ MLST primer | TGCCCTGGATCCTAATGCTCT |
| JOHE14973 | *MPD1* 3’ MLST primer | ACCCAGACTGCCGCTGTCGTC |
| JOHE15459 | *HOG1* 5’ MLST primer | AATCTGTGACTTTGGCCTTGC |
| JOHE15460 | *HOG1* 3’ MLST primer | TTCGCTGTACATCATCACCTT |
| JOHE15431 | *BWC1* 5’ MLST primer | CTCCATTCACTGCGCCAATAA |
| JOHE15432 | *BWC1* 3’ MLST primer | ATACGTGCCCTCAAAGATTCT |
| JOHE15471 | *TOR1* 5’ MLST primer | TTCGGTACCATCCTGAGTTAT |
| JOHE15472 | *TOR1* 3’ MLST primer | TTAGCCAAGGTCTTCCCACTG |
| JOHE20288 | VNTR15 5’ primer | CGGTACTGCCGATGGATAGA |
| JOHE20289 | VNTR15 3’ primer | AAAGATTTCAAGGCCCAAAGA |
| JOHE20272 | VNTR7 5’ primer | CAGCCTAATCTCACAGCCTTG |
| JOHE20273 | VNTR7 3’ primer | TCTCCCACTTCCTCGTTCAT |
| JOHE20264 | VNTR3 5’ primer | CAGAGAAGGCAAAAGGATCG |
| JOHE20265 | VNTR3 3’ primer | TCATCTTCCCCATCAGAGGT |
| MS1f | AGGAAG42 5’ primer | gaggatgttgccagagccgag |
| MS1r | AGGAAG42 3’ primer | cctcggaagatgaaatggcagc |
