# Supplementary material for: First Reported Case of Cryptococcus gattii in the Southeastern USA: Implications for Travel-Associated Acquisition of an Emerging Pathogen
Source: PLoS One. 2009 Jun 10;4(6):e5851. doi: 10.1371/journal.pone.0005851 (PMC2689935; doi:10.1371/journal.pone.0005851)
Supplement: Table S2 — (0.06 MB DOC) [file pone.0005851.s005.doc]

Table S2. Allele numbers and designations used in this study with GenBank accession numbers.

| GenBank Accession number | Submission Details |
| --- | --- |
| AY973641 | MLST allele *SXI1α*_7 |
| DQ096300 | MLST allele *SXI1*α_8 |
| DQ096304 | MLST allele *SXI1*α_12 |
| DQ096307 | MLST allele *SXI1*α_16 |
| DQ096308 | MLST allele *SXI1*α_18 |
| AY973646 | MLST allele *SXI1*α_19 |
| EU937833 | MLST allele *SXI1α*_35 |
| FJ750218* | MLST allele *SXI1α*_36 |
| AY973652 | MLST allele *SXI2***a**_1 |
| DQ096313 | MLST allele *IGS_*3 |
| DQ096314 | MLST allele *IGS_*4 |
| DQ096318 | MLST allele *IGS_*8 |
| DQ096319 | MLST allele *IGS_*10 |
| DQ096322 | MLST allele *IGS_*13 |
| DQ096324 | MLST allele *IGS_*15 |
| DQ096327 | MLST allele *IGS_*18 |
| DQ096359 | MLST allele *TEF1_*2 |
| DQ096361 | MLST allele *TEF1_*4 |
| DQ096362 | MLST allele *TEF1_*5 |
| DQ096364 | MLST allele *TEF1_*7 |
| DQ096367 | MLST allele *TEF1_*10 |
| DQ096377 | MLST allele *GPD1_*1 |
| DQ096380 | MLST allele *GPD1_*4 |
| DQ096381 | MLST allele *GPD1_*5 |
| DQ096382 | MLST allele *GPD1_*6 |
| DQ096385 | MLST allele *GPD1_*9 |
| DQ096387 | MLST allele *GPD1_*11 |
| DQ096390 | MLST allele *GPD1_*14 |
| DQ096397 | MLST allele *LAC1_*1 |
| DQ096398 | MLST allele *LAC1_*2 |
| DQ096400 | MLST allele *LAC1_*4 |
| DQ096401 | MLST allele *LAC1_*5 |
| DQ096415 | MLST allele *LAC1_*19 |
| DQ096416 | MLST allele *CAP10_*1 |
| DQ096419 | MLST allele *CAP10_*4 |
| DQ096421 | MLST allele *CAP10_*6 |
| DQ096422 | MLST allele *CAP10_*7 |
| DQ096424 | MLST allele *CAP10_*9 |
| DQ096343 | MLST allele *PLB1_*1 |
| DQ096344 | MLST allele *PLB1_*2 |
| DQ096345 | MLST allele *PLB1_*3 |
| DQ096347 | MLST allele *PLB1_*5 |
| DQ096355 | MLST allele *PLB1_*13 |
| DQ096357 | MLST allele *PLB1_*15 |
| DQ198349 | MLST allele *PLB1_*23 |
| DQ096331 | MLST allele *MPD1_*2 |
| DQ096332 | MLST allele *MPD1_*3 |
| DQ096334 | MLST allele *MPD1_*5 |
| DQ096337 | MLST allele *MPD1_*8 |
| DQ096341 | MLST allele *MPD1_*12 |
| DQ096342 | MLST allele *MPD1_*13 |
| DQ096456 | MLST allele *HOG1_*1 |
| FJ746080* | MLST allele *HOG1_*6 |
| DQ096428 | MLST allele *BWC1_*1 |
| FJ746081* | MLST allele *BWC1_*3 |
| DQ096470 | MLST allele *TOR1_*1 |
| DQ096471 | MLST allele *TOR1_*2 |
| FJ746082* | MLST allele *TOR1_*3 |
| FJ746083* | MLST allele *TOR1_*4 |
| FJ763845* | VNTR3-ST1 (WM276) |
| GQ181162* | VNTR3-ST2 (EJB1-L/A2MR314) |
| FJ763846* | VNTR7-ST1 (WM276) |
| FJ763847* | VNTR15-ST1 (WM276) |
| FJ763848* | VNTR15-ST2 (E307) |
| FJ763849* | VNTR15-ST3 (EJB1-L) |

* = Sequence first reported in this study
